# A One-Step Real-Time RT-PCR Assay for the Detection and Quantitation of *Sugarcane Streak Mosaic Virus*

**DOI:** 10.1155/2015/569131

**Published:** 2015-06-22

**Authors:** Wei-Lin Fu, Sheng-Ren Sun, Hua-Ying Fu, Ru-Kai Chen, Jin-Wei Su, San-Ji Gao

**Affiliations:** ^1^Key Laboratory of Sugarcane Biology and Genetic Breeding, Ministry of Agriculture, Fujian Agriculture and Forestry University, Fuzhou, Fujian 350002, China; ^2^College of Life Sciences, Fujian Agriculture and Forestry University, Fuzhou, Fujian 350002, China; ^3^Guangxi Collaborative Innovation Center of Sugarcane Industry, Guangxi University, Nanning, Guangxi 530005, China

## Abstract

Sugarcane mosaic disease is caused by the *Sugarcane streak mosaic virus* (SCSMV; genus *Poacevirus*, family *Potyviridae*) which is common in some Asian countries. Here, we established a protocol of a one-step real-time quantitative reverse transcription PCR (real-time qRT-PCR) using the TaqMan probe for the detection of SCSMV in sugarcane. Primers and probes were designed within the conserved region of the SCSMV coat protein (CP) gene sequences. Standard single-stranded RNA (ssRNA) generated by PCR-based gene transcripts of recombinant pGEM-CP plasmid *in vitro* and total RNA extracted from SCSMV-infected sugarcane were used as templates of qRT-PCR. We further performed a sensitivity assay to show that the detection limit of the assay was 100 copies of ssRNA and 2 pg of total RNA with good reproducibility. The values obtained were approximately 100-fold more sensitive than those of the conventional RT-PCR. A higher incidence (68.6%) of SCSMV infection was detected by qRT-PCR than that (48.6%) with conventional RT-PCR in samples showing mosaic symptoms. SCSMV-free samples were verified by infection with *Sugarcane mosaic virus* (SCMV) or *Sorghum mosaic virus* (SrMV) or a combination of both. The developed qRT-PCR assay may become an alternative molecular tool for an economical, rapid, and efficient detection and quantification of SCSMV.

## 1. Introduction

*Sugarcane mosaic virus* (SCMV),* Sorghum mosaic virus* (SrMV), and* Sugarcane streak mosaic virus* (SCSMV) are the three viruses associated with the mosaic disease of sugarcane [1]. SCMV and SrMV belong to the genus* Potyvirus*, family* Potyviridae*, while SCSMV has been recently assigned to the genus* Poacevirus* of the same family [2]. SCSMV that caused mosaic disease has been identified in many Asian countries including Pakistan, Bangladesh, Sri Lanka, Thailand, India, Vietnam, Japan, Indonesia, and China [1, 3–9]. SCSMV is the major causal pathogens of mosaic disease on sugarcane in India [10] and 10–15% yield loss has been attributed to the disease [4]. Recently, SCSMV has been noted to become widespread in the main Chinese sugarcane producing regions [9].

SCSMV viruses are rod-shaped with an average dimension of 890 × 15 nm and are similar to the members of the family* Potyviridae* [4]. The viral genome consists of a positive-sense single-stranded RNA (ssRNA) of approximately 10 kilobase (kb) in length, excluding a poly-A tail at the 3′ terminus [6, 8]. SCSMV is transmitted vegetatively during virus-infected cane cutting under natural conditions [11, 12]. In addition to sugarcane, sorghum was found to be a natural host for SCSMV [13]. Moreover, other* Poaceae* species such as maize, millet, Sudangrass, Johnson grass, crabgrass, and crowfoot grass can be infected by mechanical sap inoculation and cutting knife by SCSMV [6, 12, 14, 15].* Triticum mosaic virus* (TrMV; genus* Poacevirus*, family* Potyviridae*), which is closely related to SCSMV, is transmitted by an eriophyid mite [16], whereas the aphids* Aphis craccivora*,* Myzus persicae*, and* Rhopalosiphum maidis* serve as vectors for the potyviruses SCMV and SrMV [14]. However, a vector for the transmission for SCSMV has not yet been identified [10, 11].

SCSMV, SCMV, and SrMV cause mosaic symptoms such as interveinal chlorotic specks, streaks, or stripes on sugarcane leaves, and it is difficult to distinguish the causal virus on the basis of these symptoms alone. Moreover, the symptom expression can vary depending on age of infection, environment, and host cultivar [1]. Thus, it is important to distinguish the three viruses accurately and reliably. Many diagnostic methods are available for the detection of SCSMV in sugarcane plants including microscopic examination of viral morphology [4]; double antibody sandwich-enzyme linked immunosorbent assay (DAS-ELISA) and direct antigen coating- (DAC-)ELISA tests [17]; reverse transcription polymerase chain reaction (RT-PCR) [10]; immunocapture-RT-PCR (IC-RT-PCR) and duplex-immunocapture-RT-PCR (D-IC-RT-PCR) [18, 19]; and one-step quadruplex RT-PCR [20]. However, conventional PCR diagnostic approaches are not suitable for large scale diagnoses because they are time-consuming and have low sensitivity. Although ELISA was found to be useful for routine large scale testing of virus, it requires high quality antibodies and is limited by low sensitivity.

Hence, the development of quick and less expensive diagnostic approaches is imperative to allow for the accurate and sensitive distinction among the SCSMV, SCMV, and SrMV. More recently, the stem-loop quantitative RT-PCR (qRT-PCR) method was used to detect the miRNA encoded by the SCSMV genome [21]. The aim of the present study was to develop a real-time RT-PCR assay for the detection of SCSMV in sugarcane. Thus, our study reports a sensitive and reliable one-step TaqMan-based qRT-PCR assay for SCSMV for the first time.

## 2. Material and Method

### 2.1. Plant Material

A total of 35 of the visible top dewlap leaf samples exhibiting mosaic symptoms among commercial sugarcane cultivars (*Saccharum *spp. hybrids) were collected in 2013 in Guangdong, Guangxi, Yunnan, Hainan, Fujian, and Guizhou provinces, China. All collected leaf samples were stored at −80°C until RNA extraction.

### 2.2. RNA Extraction and Purification

Total RNA was extracted from 100 mg of sugarcane fresh leaf tissue using TRIZOL Reagent (Invitrogen/Life Technologies, Carlsbad, CA, USA) according to the manufacturer's instructions. RNase-free H_2_O (50 *μ*L) was added and then solutions were stored at −20°C. RNA was analyzed using a 1% agarose gel electrophoresis to ascertain the quality of RNA and the absence of DNA contamination. The concentration of each RNA sample was measured with micro-volume UV-Vis spectrophotometer (NanoVue Plus, GE Healthcare, Pittsburgh, PA, USA). Only the RNA samples with A260/A280 ratio (an indication of protein contamination) of 1.9–2.1 and A260/A230 ratio (an indication of reagent contamination) greater than 2.0 were used and 100 ng/*μ*L RNA was subjected to qRT-PCR and conventional RT-PCR assays.

### 2.3. Primers and Probes Design

In order to develop a real-time RT-PCR assay, primers and probes were designed according to the sequences of SCSMV CP gene published in GenBank library using the Primer Express software version 2.0 (ABI Applied Biosystems, Foster City, CA, USA). For conventional RT-PCR detection of SCSMV, a set of primers SCSMV-CPF2 and SCSMV-CPR2 were designed to correspond with the 572 base pair (bp) fragment of SCSMV CP gene. All primers and probes were targeted in the conserved region within the fragment. The TaqMan probes were labeled with 6-carboxy-fluorescein (FAM, excitation wavelength 494 nm, emission wavelength 521 nm) reporter dye at 5′-end and 6-carboxytetramethylrhodamine (TAMRA) fluorescent quencher at the 3′-end. The details of all primers and probes are listed in Table 1.

### 2.4. RNA Transcripts of SCSMV CP Synthesis* In Vitro*

SCSMV CP fragment (572 bp) was amplified with SCSMV-CPF2 and SCSMV-CPR2 from YN-YZ211 isolates (GenBank acc. no. KJ187047) and inserted into the pGEM-T Easy (Promega, Madison, WI, USA) and then it transformed the plasmid into competent cell of* Escherichia coli* strain DH5*α*. The right inserted PCR product was verified by sequencing. Positive-sense single-strand RNA (ssRNA) was transcribed using the RiboMAX Large Scale RNA Production Systems-T7 Kit (Promega), using 2 *μ*L linearized recombinant plasmid DNA (1 *μ*g) as the template, and then the plasmid DNA was digested with RNase-free DNase I at 37°C for 20 min. The RNA was purified with RNAclean kit (BioTeke, Beijing, China) and then was quantified using NanoVue Plus.

### 2.5. One-Step Real-Time qRT-PCR Assay

One-step real-time qRT-PCR assay was performed with the final volume of 20 *μ*L using the one-step PrimeScript RT-PCR Kit (TaKaRa Biotech, Dalian, China) following the manufacturer's instructions. Each reaction was carried out using 1 *μ*L of ssRNA transcripts from pGEM-CP plasmid or 2 *μ*L of total RNA extracted from collected samples as template on the ABI 7500 Real-Time PCR Detection System (Applied Biosystems, USA). During the amplification process, the fluorescence intensity of the reporter dye (FAM) and a quencher dye (TAMRA) were recorded. These data allowed the calculation of the normalized reporter signal, which is linked to the amount of product amplified. The threshold cycle (Ct values, number of amplification cycles for the fluorescence to reach the threshold) refers to the number of amplification cycles required for a significant increase in the reporter's fluorescence. The data were analyzed with ABI 7500 Real-Time PCR Detection System program.

Upstream and downstream primers were subjected to a 3 × 3 optimization matrix using a concentration of 100, 200, and 400 nmol/*μ*L for each concentration of primer under 200 nmol/*μ*L probe concentration. Subsequently, the concentration of the TaqMan probe was optimized. ssRNA transcripts synthesized* in vitro* were used as template. The most suitable program and parameter were reverse transcription of the RNA at 42°C for 5 min. The optimum denaturation time was 10 sec at 95°C. Annealing-extension time and temperature were 34 sec at 60°C.

A standard curve was generated using tenfold serial dilutions ranging from 10^9^ to 10^2^ copies of ssRNA transcripts or 100 ng to 1 pg of total RNA extracted from sugarcane leaf with SCSMV-infected YN-YZ211 isolate. Ct values were measured in three duplicates and plotted against the known copy numbers of the standard samples. These standard curves were determined to estimate the reaction efficiency and the quantification of viral target in the unknown samples. The reaction efficiency under our experimental conditions was addressed as % and calculated with the formula *E* = (10^−1/slope^ − 1) × 100%.

### 2.6. Conventional RT-PCR

In parallel, the same set of tenfold serial dilutions of ssRNA and total RNA were carried out by conventional RT-PCR assay using SCSMV-CPF2 and SCSMV-CPR2 primers for sensitivity tests. SCSMV was detected in the field samples using the abovementioned primers. In addition to this primer pair, two other sets of primers, SCMV-F4 and SCMV-R3 published by Alegria et al. (2003) [22] and SrMV-F and SrMV-R reported by Xie et al. (2009) [20], were used for screening the SCMV and SrMV, respectively. PCR amplification was performed in a total volume of 25 *μ*L containing 2 *μ*L RNA with 1 *μ*L PrimeScript one-step Enzyme Mix (TaKaRa, shanghai, China), 400 nmol/L of each of the primers, and 200 *μ*mol/L of dNTPs mixture. The RT-PCR program was reverse transcription of RNA at 50°C for 30 min, and PCR performed at 94°C 2 min, 35 cycles at 94°C 30 sec, 55°C for SCSMV or 52°C for SrMV or 65°C for SCMV 30 sec, and 72°C 1 min, followed by a final 72°C extension step for 5 min. The RT-PCR amplified products were detected on a 1.5% agarose gel and then cloned into pMD19-T vectors (TaKaRa, Dalian, China) for sequencing.

### 2.7. Sequence Analysis

SCSMV, SrMV, and SCMV sequences obtained with individual primers were analyzed by BLAST search in NCBI (http://blast.ncbi.nlm.nih.gov/). Alignment of the identified CP sequence (572 nt) from SCSMV YN-YZ211 isolate and other 24 SCSMV sequences published in GenBank were performed using MUSCLE algorithm [23] implemented in MEGA software program version 5 [24]. Phylogenetic tree was generated by the neighbor-joining method with 1,000 bootstrap replications using MEGA 5.

## 3. Results

### 3.1. Sequence Alignment and Primer Specificity

**Figure 1:**
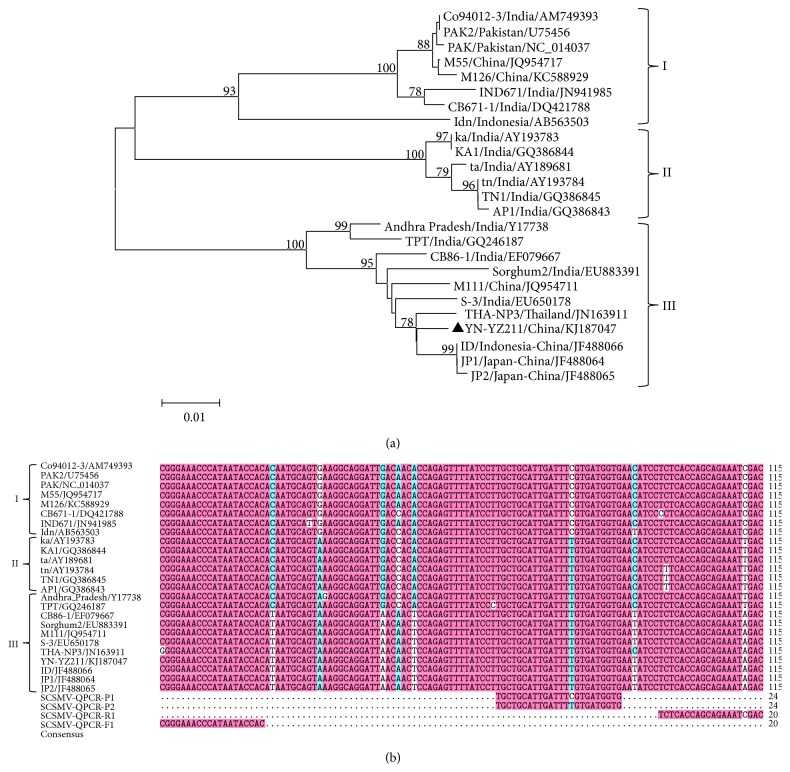
(a) Phylogenetic tree derived from the CP nucleotide sequences (572 nt) of* Sugarcane streak mosaic virus* (SCSMV). The tree was generated using MEGA 5 by the neighbor-joining method with 1,000 bootstrap replicates. The bootstrap confidence values are indicated when >60%. The NCBI accession number of the sequences used in the comparison is shown along with isolates' name and the isolates from this study were marked with a black triangle. (b) Primers and probes sequences used in qRT-PCR assay aligned with the same set of CP sequences by DNAMAN version 8.

The phylogenetic tree of the SCSMV CP or P1 sequences was used to report the three SCSMV genotypes from Asian countries [9, 10]. In the present study, three SCSMV genotypes (I, II, and III) were also clustered by the phylogenetic tree based on the CP nucleotide (572 nt) sequences from YN-YZ211 isolate and 24 representative isolates published in the GenBank database (Figure 1(a)). Two SCSMV genotypes (SCSMV-I and -III) from the Chinese isolates were observed, and YN-YZ211 isolate was grouped as the SCSMV-III genotype. All the Indian isolates were clustered into the SCSMV-II genotype. The real-time PCR primers and probes were designed based on a highly conserved region of the SCSMV CP sequences. The primers and probes specificities were evaluated based on multiple sequence alignment (Figure 1(b)). Sense primer of SCSMV-QPCR-F1 was completely identified with the sequences of three SCSMV genotypes while the antisense primer of SCSMV-QPCR-R1 had one variable nucleotide (A/C/T) positioned at the 5′ end of the primer. One variable nucleotide (C/T) was located in the middle of the probe region. Therefore, the two specific probes were designed for developing a TaqMan-based real-time qRT-PCR in this study.

### 3.2. Standard Curve of the Real-Time qRT-PCR Assays

The optimum concentration for both upstream and downstream primers of SCSMV real-time RT-PCR was found to be 200 nmol/*μ*L. The concentration of the TaqMan probe (SCSMV-QPCR-P1 or -P2) was optimized as 200 nmol/*μ*L. ssRNA was synthesized through RNA transcripts of pGEM-CP* in vitro* to obtain the standard curve for the real-time qRT-PCR assays. The standard curves were established by using tenfold serial dilutions of the standard ssRNA ranging from 10^2^ to 10^9^ copies to determine the end-point limit of detection and for the one-step real-time qRT-PCR assays. Furthermore, the total RNA extracted from the sugarcane leaf was infected with the SCSMV YN-YZ211 isolate and diluted to a tenfold serial solution (1 pg/*μ*L to 100 ng/*μ*L) for establishing the standard curves. Ct values (three replicates) were measured and plotted against the known copy numbers of the standard samples. The standard curves were generated by two different probes, SCSMV-QPCR-P1 and SCSMV-QPCR-P2.

**Figure 2:**
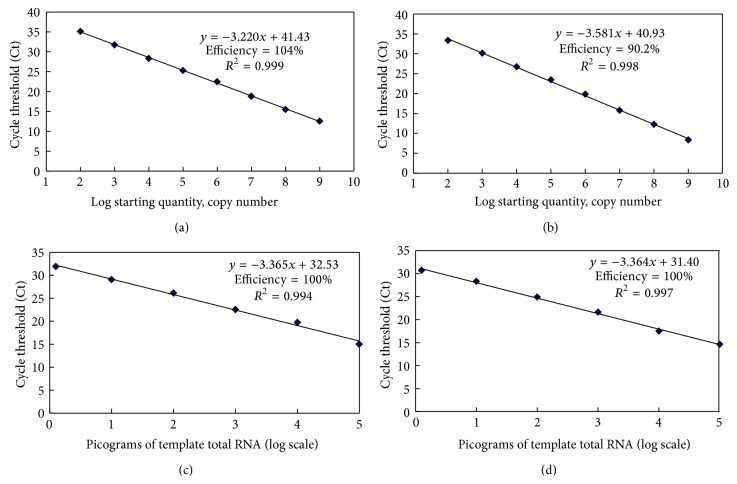
Standard curves for TaqMan probes-based real-time qRT-PCR using SCSMV CP transcripts (positive-sense single-stranded RNA, ssRNA) ((a) and (b)) and total RNA ((c) and (d)) of plant infected SCSMV YN-YZ211 isolate as templates. A tenfold dilution serial standard template containing ssRNA ranged from 1 × 10^9^ to 1 × 10^2^ copies/reaction. Total RNA templates ranged from 2 pg to 200 ng/reaction. Ct values were measured in triplicate and plotted against the known copy numbers (log scale) or picograms of template total RNA (log scale). Two different probes, SCSMV-QPCR-P1 ((a), (c)) and SCSMV-QPCR-P2 ((b), (d)), were developed.

Standard curves together with the reaction efficiencies and slopes are shown in Figure 2. For ssRNA as template, the standard curve covered a linear range of eight orders of magnitude (Figures 2(a) and 2(b)). The slopes from the standard curve of SCSMV-QPCR-P1 and SCSMV-QPCR-P2 probes were −3.220 and −3.581, efficiency (*E*, %) = 104% and 90.2%, and *R* ^2^ = 0.999 and 0.998, respectively. For total RNA as template, the standard curves covered a linear range of six orders of magnitude (Figures 2(c) and 2(d)). We observed that the standard curve for SCSMV-QPCR-P1 and SCSMV-QPCR-P2 probes had similar slopes of −3.365 and −3.364 and *R* ^2^ = 0.994 and 0.997, respectively. Both standard curves showed the same efficiency of 100%. This demonstrated that the TaqMan real-time RT-PCR protocol was feasible for the quantification of the SCSMV pathogen.

### 3.3. Assessment of the Real-Time qRT-PCR Assays

**Figure 3:**
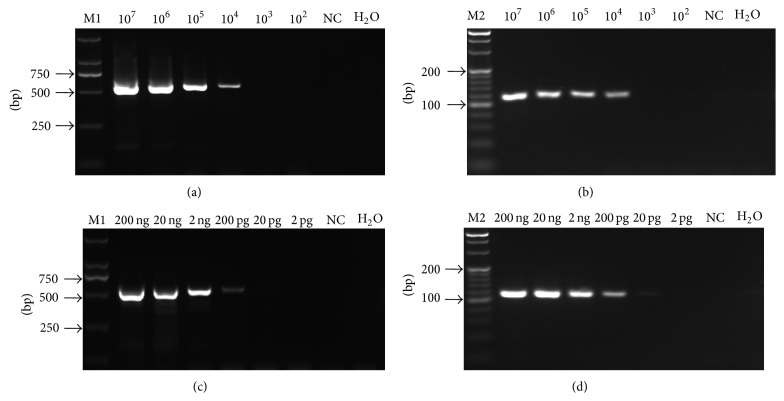
Gel-based sensitivity tested using the two set primers of qRT-PCR (SCSMV-QPCR-F1 and -R1) ((a) and (c)) and conventional RT-PCR (SCSMV-CPF2 and -CPR2) ((b) and (d)). The same set of templates (10^7^–10^2^ copies/reaction) of SCSMV CP transcripts (positive-sense single-stranded RNA, ssRNA) was used in (a) and (b), and the same set of diluted solutions (2 pg to 200 ng/reaction) of total RNA extraction of sugarcane leaf infected SCSMV YN-YZ211 isolate were carried out in (c) and (d). M1. DL 2,000 DNA marker; M2. 20 bp DNA ladder marker; NC. Virus-free total RNA (100 ng).

In order to compare the end-point sensitivity between TaqMan qRT-PCR and conventional RT-PCR assays, the same set of templates of six tenfold serial dilutions (10^7^–10^2^ copies) of standard ssRNA were used in conventional RT-PCR with two pairs of primers (SCSMV-CPF2 and -CPR2 and SCSMV-QPCR-F1 and -R1). The expected amplicons of 572 bp and 115 bp were obtained from the reaction, which were confirmed by agarose gel electrophoresis. The detection limits were 1 × 10^4^ copies for each set of primers (Figures 3(a) and 3(b)). However, we observed a titer as low as 1 × 10^2^ copies of standard ssRNA in a one-step TaqMan real-time RT-PCR assay (Figures 2(a) and 2(b)). Thus, our results suggested that the real-time qRT-PCR was 100-fold more sensitive than the conventional RT-PCR.

The same set of templates, that is, the six tenfold dilutions (from 100 ng/*μ*L to 1 pg/*μ*L) of total RNA, were also used in the conventional RT-PCR. Figures 3(c) and 3(d) present the typical data from a serial dilution of total RNA extracted from the SCSMV-infected leaf with a detection limit of 200 pg using either of the primer pairs, that is, SCSMV-CPF2 and -CPR2 and SCSMV-QPCR-F1 and -R1. However, the minimum detection limit of real-time RT-PCR assay was as low as 2 pg (Figures 2(b) and 2(d)). We deduced that more than 100-fold sensitivity in the one-step TaqMan-based real-time qRT-PCR assay compared with the conventional RT-PCR.

### 3.4. Application of Real-Time RT-PCR Assays

A total of 35 sugarcane samples with mosaic symptoms were collected from the sugarcane fields in China for determination of SCSMV infection using both one-step real-time qRT-PCR and conventional RT-PCR assays. Among the 35 samples, qRT-PCR identified 68.6% (24/35) positives while only 48.6% (17/35) of samples were detected as positive by the conventional RT-PCR assay (Table 2). The virus titer of SCSMV-infected field samples ranged from 10^2^ to 10^6^ copies/*μ*L. To rule out false negatives in SCSMV infection, in addition to using SCSMV-uninfected sugarcane sample as negative control, SCSMV-infected sample and water were also used as positive and blank controls. Furthermore, all of the RNA samples were checked for SCMV and SrMV detection using conventional RT-PCR assay. We found that 11 SCSMV-free samples, which were verified by qRT-PCR and conventional RT-PCR, were infected with either SCMV or SrMV, or both. On the other hand, to exclude nonspecific amplification, all qRT-PCR or conventional RT-PCR products were verified by sequencing, and their nucleotide sequences were compared using the BLAST tool (http://blast.ncbi.nlm.nih.gov/). These findings indicated that qRT-PCR yields more accurate results and helps detect more positive samples in a low virus titer as compared with the conventional gel-based RT-PCR.

## 4. Discussion

Mosaic disease of sugarcane caused by SCSMV has been reported in Asian countries including India and China. The SCSMV has become widespread in the sugarcane-growing fields in China [9]. Three SCSMV genotypes have been identified in China and India based on the phylogenetic trees of the P1 or CP sequences [9, 10]. All 17 SCSMV isolates identified in the present study by conventional RT-PCR shared high nucleotide sequence identity (97.7–99.8%) and were grouped under the SCSMV-III genotype (Table 2). A representative isolate, YN-YZ211, was used as a positive control. The disease caused by SCSMV is difficult to distinguish from those caused by SCMV and SrMV because of similar symptoms and occasional coinfection with the two viruses. RT-PCR, reverse transcription loop-mediated isothermal amplification (RT-LAMP), and serological methods are most commonly used for detecting sugarcane pathogens; however, these methods do not effectively distinguish among the causal pathogens. In the present study, a one-step qRT-PCR assay, using TaqMan, was established for the quick detection and quantification of SCSMV in sugarcane plants. This assay yielded highly sensitive results by using a fluorescence signal.

The specificity of the one-step qRT-PCR assay has been evaluated based on the multiple sequence alignment. Although we found few nucleotides in 5′ end of primers varied among the published SCSMV genotypes, this mismatch would not significantly affect the PCR efficiency. Besides, one nucleotide varied in probe sequence region between SCSMV genotypes; therefore, two different probes (SCSMV-QPCR-P1 and -P2) were designed to assess the differences of PCR efficiency with the same RNA template. A permissible difference of PCR efficiency (104% for SCSMV-QPCR-P1 and 90.2% for SCSMV-QPCR-P2) was presented for ssRNA template (Figures 2(a) and 2(b)); on the other hand, no difference of PCR efficiency was found between the two probes for total RNA template (Figures 2(c) and 2(d)). SCSMV-QPCR-P2 was used as a probe for determination of SCSMV infection in the field samples (as it is showed in Table 2) as its sequence was identified to positive control of ssRNA template of YN-YZ211 isolate. This developed method in this study can be effectively applied in SCSMV diagnoses. Moreover, none of the other sugarcane causal viruses, SCMV, SrMV, and* Sugarcane yellow leaf virus* (SCYLV), were detected using the primers and probes of this qRT-PCR assay (data not shown).

In real-time quantification of PCR assay, the Ct value is a parameter reflecting the quantity of templates presented in the reaction [25]. As always, the concentration of the template was linear in correlation to the Ct values. The efficiency (slope) and the coefficient of determination (*R* ^2^) of the linear equation were determined to optimize the concentrations and conditions of the real-time RT-PCR assay. The standard serial dilution curve was obtained with a rational qRT-PCR efficiency for SCSMV (90.2–104%) in this study using either ssRNA transcripted* in vitro* or plant total RNA as template. Besides, the *R* ^2^ values of SCSMV were very close to 1 (0.994–0.999). Two specific probes designed in this study can be used in the TaqMan-based real-time RT-PCR for SCSMV. Therefore, this qRT-PCR assay could be used for the quantitative detection of SCSMV in routine virus diagnoses.

High sensitivity is one of the salient features of qRT-PCR. In this assay, a minimum titer of SCSMV could be determined for as low as 100 copies of ssRNA or 2 pg total RNA. qRT-PCR was at least 100-fold more sensitive compared with conventional RT-PCR techniques. Furthermore, higher sensitivity of qRT-PCR was observed in SCSMV detection of sugarcane field samples. Similar sensitivity results were found in SCYLV detection with TaqMan probe-based qRT-PCR assay [26, 27]. The nonspecific amplification in SCSMV qRT-PCR assay was excluded because all qRT-PCR products were further verified by sequencing. Similarly, false negatives for the qRT-PCR assay were ruled out by including RNA from SCSMV-infected and SCSMV-uninfected samples as positive and negative controls, respectively. Water was used as a blank control. Moreover, the samples with mosaic symptom could not be determined by SCSMV qRT-PCR or regular RT-PCR but were further detected by infection with SCMV or SrMV or both. The virus titers of SCSMV infecting field samples ranged from 10^2^ to 10^6^ copies/*μ*L. It is worthy noted that the negative SCSMV samples might be false negatives in the case of the virus titre in leaf samples less than the minimum detection limit of qRT-PCR or conventional RT-PCR assays.

SCSMV is mainly transmitted through virus-infected cane cuttings among sugarcane-planting countries and regions. Therefore, it is necessary to develop a specific, rapid, and sensitive detection method for screening germplasms in quarantine and determine the distribution of the SCSMV in commercial fields. A reliable diagnosis is vital for the development of efficient strategies for viral disease control. Therefore, our study reports a highly specific, sensitive, and reliable qRT-PCR assay using TaqMan probe for the detection and quantification of SCSMV in sugarcane.

## 5. Conclusion

In this study, we firstly established a protocol of TaqMan probe-based real-time qRT-PCR for the detection and quantification of SCSMV causing sugarcane mosaic disease. This qRT-PCR assay is an optional molecular tool to be used in a rapid, sensitive, and efficient diagnosis of SCSMV in sugarcane quarantine and disease control.

## Figures and Tables

**Table 1:** List of the primers and probes used in this study.

Virus	Primer name	Sequence (5′-3′)	Fragment size (bp)
SCSMV	SCSMV-CPF2	TCATMTCTTCATCRGCCGC	572
	SCSMV-CPR2	ATCTTCYCTACGCAGGTCCG	
	SCSMV-QPCR-F1	CGGGAAACCCATAATACCAC	115
	SCSMV-QPCR-R1	GTCGATTTCTGCTGGTGAGA	
	SCSMV-QPCR-P1	FAM-TGCTGCATTGATTT**C**GTGATGGTG-TAMRA	
	SCSMV-QPCR-P2	FAM-TGCTGCATTGATTT**T**GTGATGGTG-TAMRA	
SCMV	SCMV-F4	GTTTTYCACCAAGCTGGAACAGTC	900
	SCMV-R3	AGCTGTGTGTCTCTCTGTATTCTC	
SrMV	SrMV-F	ACAGCAGAWGCAACRGCACAAGC	850
	SrMV-R	CTCWCCGACATTCCCATCCAAGCC	

SCMV-F4/SCMV-R3 and SrMV-F/SrMV-R published in Alegria et al. [22] and Xie et al. [20], respectively.

M = A/C, Y = C/T, W = A/T, R = A/G, and W = A/T in primer sequences.

FAM: 6-carboxyfluorescein, TAMRA: 6-carboxy tetramethyl rhodamine.

**Table 2:** Detection of SCSMV from field sugarcane samples by real-time qRT-PCR with SCSMV-QPCR-P2 probe and conventional RT-PCR.

Varieties	Sampling location	qRT-PCR			Conventional RT-PCR		
		Ct value	Copies/*μ*L	Result	SCSMV	SrMV	SCMV
FN38	Fuzhou, Fujian	30.53 ± 0.28	8.2 × 10^2^	+	—	+, [KC984957]	—
FN15	Fuzhou, Fujian	na	na	−	—	+, [KC984956]	—
ROC22	Fuzhou, Fujian	30.37 ± 0.16	8.9 × 10^2^	+	—	+, [KC984964]	+, [KC899320]
FN39	Zhangzhou, Fujian	na	na	−	—	+, [KC984958]	—
ROC22	Guangzhou, Guangdong	na	na	−	—	+, [KC984980]	—
F160	Suixi, Guangdong	20.51 ± 0.33	5.1 × 10^5^	+	+, [KJ944725]	+, [KJ944744]	—
GT31	Suixi, Guangdong	33.51 ± 0.34	1.2 × 10^2^	+	+, [KJ944729]	+, [KJ944745]	—
LC05-136	Suixi, Guangdong	na	na	−	—	+, [KJ944746]	—
ROC22	Suixi, Guangdong	32.68 ± 0.42	2.0 × 10^2^	+	+, [KJ944727]	+, [KJ944741]	—
YZ05-49	Suixi, Guangdong	19.34 ± 0.14	1.1 × 10^6^	+	+, [KJ944728]	+, [KJ944742]	—
YZ07-2384	Zhanjiang, Guangdong	16.72 ± 0.23	5.8 × 10^6^	+	+, [KJ944726]	+, [KJ944736]	—
GT11	Wangmo, Guizhou	32.55 ± 0.30	2.2 × 10^2^	+	+, [KC985070]	+, [KC985029]	—
GZ18	Wangmo, Guizhou	31.39 ± 0.5	4.6 × 10^2^	+	+, [KC985071]	—	—
YZ03-45	Wangmo, Guizhou	32.21 ± 0.15	2.7 × 10^2^	+	+, [KC985072]	+, [KC985030]	—
YG35	Kaiyuan, Yunnan	18.42 ± 0.36	1.9 × 10^6^	+	+, [KC985085]	+, [KC985056]	—
YN01-58	Kaiyuan, Yunnan	31.26 ± 0.25	5.0 × 10^2^	+	+, [KC985086]	+, [KC985061]	—
YZ05-211	Kaiyuan, Yunnan	21.19 ± 0.29	3.3 × 10^5^	+	+, [KC985090]	+, [KC985064]	—
YT93-159	Lincang, Yunnan	32.59 ± 0.31	2.1 × 10^2^	+	—	+, [KC985060]	—
FN15	Danzhou, Hainan	32.35 ± 0.22	2.5 × 10^2^	+	—	+, [KC985031]	—
YT55	Danzhou, Hainan	31.52 ± 0.27	4.2 × 10^2^	+	—	+, [KC985038]	—
ROC22	Haikou, Hainan	32.07 ± 0.07	3.0 × 10^2^	+	+, [KC985074]	—	—
YZ05-49	Lingao, Hainan	16.44 ± 0.27	6.9 × 10^6^	+	+, [KC985075]	+, [KC985041]	—
GT02-281	Beihai, Guangxi	32.13 ± 0.19	2.9 × 10^2^	+	+, [KC985069]	+, [KC984999]	—
FN1110	Bingyang, Guangxi	na	na	−	—	+, [KJ944748]	—
YT93-159	Bingyang, Guangxi	na	na	−	—	+, [KJ944750]	+, [KJ944720]
FN39	Laibin, Guangxi	32.27 ± 0.32	2.6 × 10^2^	+	+, [KJ944735]	+, [KJ944782]	—
ROC16	Laibin, Guangxi	32.17 ± 0.21	2.8 × 10^2^	+	—	+, [KJ944757]	—
ROC22	Laibin, Guangxi	29.60 ± 0.12	1.5 × 10^3^	+	+, [KJ944731]	+, [KJ944764]	—
YG46	Laibin, Guangxi	32.92 ± 0.35	1.7 × 10^2^	+	+, [KJ944734]	+, [KJ944774]	—
YR06-189	Laibin, Guangxi	32.89 ± 0.46	1.8 × 10^2^	+	—	+, [KJ944758]	—
GT05-3084	Longan, Guangxi,	na	na	−	—	+, [KJ944820]	—
GT07-994	Liuzhou, Guangxi	na	na	−	—	+, [KJ944814]	—
ROC22	Liuzhou, Guangxi	na	na	−	—	+, [KJ944815]	—
FN15	Shanglin, Guangxi	na	na	−	—	+, [KJ944752]	—
ROC22	Shanglin, Guangxi	na	na	−	—	+, [KJ944754]	—

Positive result (+) if Ct ≤ 35, negative result (−) if Ct > 35.

GenBank acc. no. of these sequences from conventional RT-PCR product were in brackets.

na: data not available.
